# The Infectious Tale of Aerococcal Aortic Endocarditis: Cardiac Pacing in Acute Aortic Insufficiency With Complete Heart Block as a Bridge to Aortic Valve Replacement

**DOI:** 10.7759/cureus.17921

**Published:** 2021-09-13

**Authors:** Laura Bradel, Kartikeya Kashyap, Fouad Jabbour, Pietro Bajona, Victor Farah, Vinh Nguyen

**Affiliations:** 1 Internal Medicine, Arnot Ogden Medical Center, Elmira, USA; 2 Cardiology, Allegheny Health Network, Pittsburgh, USA; 3 Cardiothoracic Surgery, Allegheny Health Network, Pittsburgh, USA

**Keywords:** surgical aortic valve replacement (savr), aortic valve abscess, aortic insufficiency, aerococcus infective endocarditis, complete heart block, gated coronary computed tomography, cardiac pacing

## Abstract

Here, we describe a rare case of *Aerococcus* endocarditis causing aortic insufficiency and paravalvular abscess presenting as complete heart block and shock. A 76-year-old man with diabetes mellitus presented to the emergency department with fever and dyspnea. His temperature was 102.4°F, heart rate 59 beats per minute, blood pressure 105/44 mmHg, and oxygen saturation was 98% on 6L oxygen. Examination revealed bounding carotid pulses, a 2/6 early blowing diastolic murmur at the left lower sternal border, and diminished lung sounds at the bases. Laboratory data showed leukocytosis of 19.65 k/µL, blood urea nitrogen 72 mg/dL, creatinine 2.92 mg/dL, lactic acid 3.1 mmol/L, pro-B-type natriuretic peptide 15,342 pg/mL, high-sensitivity troponin 136 ng/L, aspartate aminotransferase 129 U/L, and alanine aminotransferase of 115 U/L. An electrocardiogram showed complete heart block, and a transvenous pacemaker was placed. A transesophageal echocardiogram revealed an aortic root abscess and severe aortic insufficiency secondary to *Aerococcus urinae*. Ventricular pacing was used to decrease aortic insufficiency and optimize computed tomography with gating to view the coronary arteries due to wall motion abnormalities seen on the transthoracic echocardiogram. His aortic valve was replaced, and a pacemaker was planned. Aortic valve *Aerococcus* endocarditis is rare and can lead to complete heart block and aortic insufficiency. Cardiac pacing improves hemodynamics by increasing heart rate and decreasing left ventricular end-diastolic pressure.

## Introduction

*Aerococcus* infectious endocarditis of a native aortic valve is extremely rare [1]. The infection occurs in elderly males with underlying urological abnormalities with an estimated prevalence of endocarditis of 54/1,000,000 [2-4]. In this case report, we describe a case with severe aortic insufficiency and paravalvular abscess with pseudoaneurysm presenting as heart block and shock.

This article was previously presented as an abstract at the Society for Vascular Medicine 2021 Scientific Conference held on September 9-11, 2021 and will be published in *Vascular Medicine*.

## Case presentation

**Figure 1:**
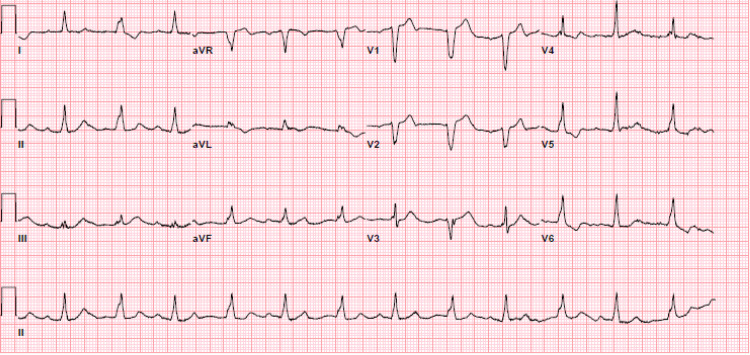
Electrocardiogram showing complete heart block with junctional escape.

A 76-year-old man with chronic obstructive pulmonary disease and type II diabetes mellitus presented to the emergency department with fever, dyspnea on exertion, and lethargy. At presentation, his temperature was 102.4°F, heart rate 59 beats per minute, blood pressure 105/44 mmHg, and oxygen saturation was 98% on a 6 L nasal cannula. Examination revealed bounding carotid pulses, a grade 2/6 early blowing diastolic murmur at the left lower sternal border, and diminished lung sounds at the bases. Laboratory profile showed leukocytosis of 19.65 k/µL, acute-on-chronic kidney injury with blood urea nitrogen of 72 mg/dL and creatinine of 2.92 mg/dL, lactic acid 3.1 mmol/L, pro-B-type natriuretic peptide 15,342 pg/mL, high-sensitivity troponin 136 ng/L, aspartate aminotransferase 129 U/L, and alanine aminotransferase of 115 U/L. Serial electrocardiogram showed complete heart block with alternating bundle branch morphology and junctional escape (Figure 1), requiring a transvenous pacemaker.

A transthoracic echocardiogram showed normal biventricular function, moderate aortic insufficiency with no evidence of aortic abscess, and hypokinetic mid-distal anteroseptal segments. Cardiac pacing was set to 100 beats per minute to limit diastolic time and reduce aortic insufficiency. Despite aggressive therapy with broad-spectrum antibiotics including vancomycin and cefepime for less than two days, he decompensated and required emergent intubation and was placed on epinephrine and vasopressin. Blood cultures grew *Aerococcus urinae*. He developed methicillin-sensitive *Staphylococcus aureus* ventilator-associated pneumonia. Antibiotics were de-escalated and he was treated with a 14-day course of ampicillin-sulbactam.

**Figure 2:**
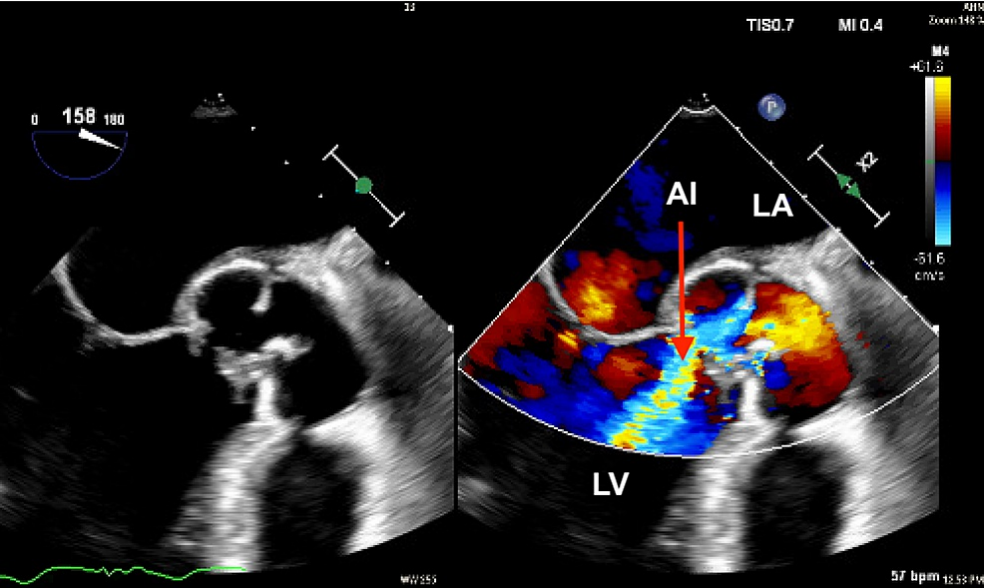
Transesophageal echocardiographic findings of aortic root abscess with severe aortic insufficiency (red arrow). AI: aortic insufficiency; LV: left ventricle; LA: left atrium

A transesophageal echocardiogram revealed a 1.7 cm × 2.7 cm paravalvular aortic root abscess occupying the noncoronary sinus and left ventricular outflow tract with severe aortic regurgitation (Figure 2).

**Figure 3:**
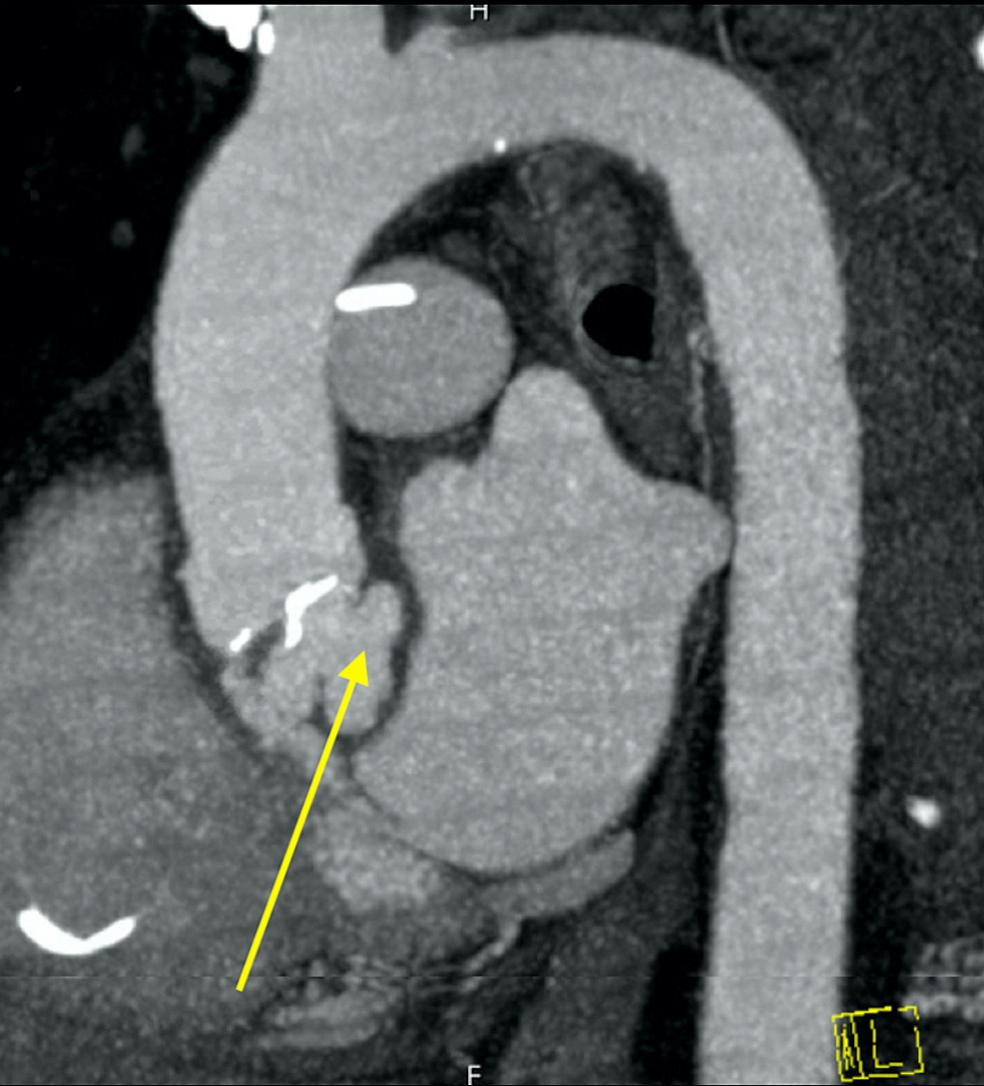
Gated coronary computed tomography with further visualization of aortic root abscess (yellow arrow).

**Figure 4:**
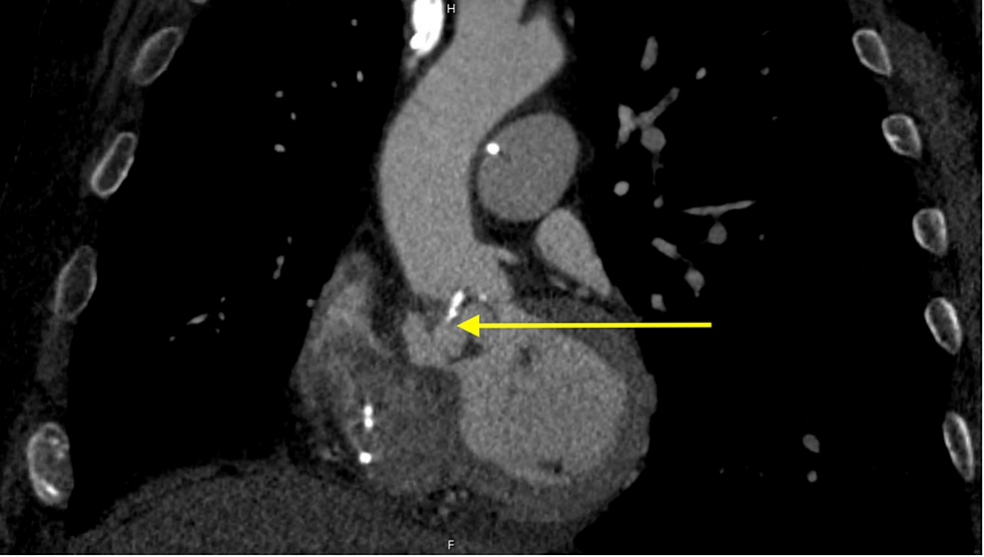
Gated coronary computed tomography showing aortic root abscess (yellow arrow).

Computed tomography (CT) of the chest, abdomen, and pelvis was performed to identify a potential infectious source. Due to wall motion abnormalities and concern for coronary embolization, the study was performed with gating, and ventricular pacing was decreased to 50 beats per minute to optimize coronary artery visualization (Figures 3, 4).

CT confirmed the abscess with accompanying subaortic pseudoaneurysm along the aorto-mitral curtain, pyelitis, and cystitis. No acute coronary artery pathology was identified. The patient underwent emergent aortic valve replacement, debridement of subaortic pseudoaneurysm, and bovine pericardial patch repair of the aorta (Figure 4). After the pneumonia was treated, he was treated with penicillin G for six weeks from the time of the aortic valve surgery. His postoperative hemodynamics improved, and a leadless pacemaker was planned when blood cultures cleared.

**Figure 5:**
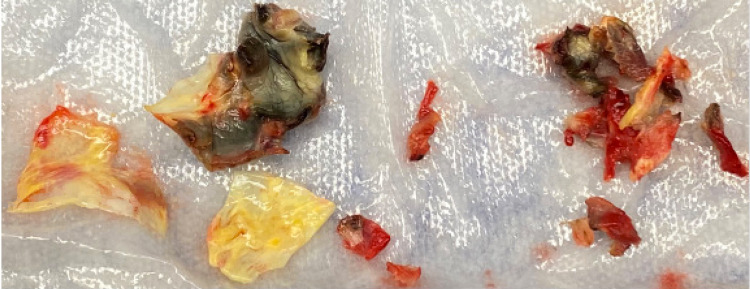
Pathological specimen of the aortic root abscess.

## Discussion

The occurrence of *Aerococcus urinae* endocarditis is rare [4]. Risk factors include males over the age of 65, with predisposing factors such as diabetes mellitus, ischemic heart disease, tumors, and urological tract pathology [4,5]. Common complications of endocarditis include intracardiac abscess and new conduction abnormalities with peri-annular extension, as seen in this patient [6]. The patient presented with a rare case of aerococcal native aortic valve endocarditis complicated by aortic root abscess leading to aortic insufficiency, complete heart block, and cardiogenic shock. Class I indications for surgery among patients with infective endocarditis independent of the completion of a full therapeutic course of antibiotics include valve destruction leading to symptoms of heart failure, left-sided infectious endocarditis caused by highly resistant microorganisms, complications of heart block, annular or aortic abscess, destructive lesions, and persistent bacteremia lasting more than seven days after starting appropriate antibiotic therapy [7]. In this case, radical debridement and reconstruction of the root with patch reconstruction and valve replacement were the cornerstones of therapy, and early surgery remained the key for survival.

Bridging therapy until definitive surgical management can include cardiac pacing and vasopressor support. Due to the normal ventricle size in acute aortic regurgitation, the regurgitant volume causes an acute increase in end-diastolic pressure and impairs forward stroke volume [8,9]. Intra-aortic balloon counter-pulsation is contraindicated as it can worsen aortic regurgitation due to balloon inflation in diastole [10]. Bradycardia can lead to pulmonary edema and secondary prolonged diastolic time [11]. Increasing the heart rate can improve the overall hemodynamics by decreasing the left ventricular end-diastolic pressure and pulmonary arterial wedged pressure, and by increasing the cardiac index by decreasing the time in diastole [4]. This can be achieved through cardiac pacing with the most favorable hemodynamics at heart rates between 110 and 130 beats per minute [11].

## Conclusions

*Aerococcus urinae* disseminated infections are rare with infective endocarditis being the most common. Early surgical valve replacement is key to survival among high-risk patients. In acute severe aortic regurgitation, medical therapy in conjunction with cardiac pacing can be used to reduce left ventricular afterload and increase cardiac output for temporarily stabilizing the patient until definitive surgical management.
